# SARS-CoV-2 infection of the nervous system: A review of the literature on neurological involvement in novel coronavirus disease-(COVID-19)

**DOI:** 10.17305/bjbms.2020.4860

**Published:** 2020-08

**Authors:** Alvin Oliver Payus, Constance Liew Sat Lin, Malehah Mohd Noh, Mohammad Saffree Jeffree, Raymond Azman Ali

**Affiliations:** 1 Faculty of Medicine and Health Science, Universiti Malaysia Sabah (UMS), Kota Kinabalu, Sabah, Malaysia; 2 Department of Internal Medicine, Universiti Kebangsaan Malaysia Medical Centre (UKMMC), Cheras, Kuala Lumpur, Malaysia

**Keywords:** Coronavirus disease 2019, COVID-19, coronavirus, severe acute respiratory syndrome coronavirus 2, SARS-CoV-2, pandemic, nervous system, central nervous system, CNS, peripheral nervous system, PNS

## Abstract

The novel coronavirus disease 2019 (COVID-19) is caused by the severe acute respiratory syndrome coronavirus 2 (SARS-CoV-2), which is believed to have emerged from an animal source and has been spreading rapidly among humans. Recent evidence shows that SARS-CoV-2 exhibits neurotropic properties and causes neurological diseases. Here, we review the literature on neurological involvement in SARS-CoV-2 infections and the possible mechanisms of invasion of the nervous system by this virus, to provide a summary and critical analysis of the early reporting of neurological involvement in COVID-19. An exhaustive search of scientific articles on neurological involvement in COVID-19 was performed in the Web of Science, Scopus, Medline/PubMed, and several other databases. Nineteen relevant articles that had been published or were in preprint were carefully selected according to the inclusion and exclusion criteria. Based on our research, we found that patients with COVID-19 can present with neurological symptoms that can be broadly divided into central nervous system involvement, such as headache, dizziness, altered mental state, and disorientation, and peripheral nervous system involvement, such as anosmia and hypogeusia. Most of these patients are in the older age group and exhibit comorbidities, especially hypertension, and severe infection. In extreme presentations of COVID-19, some patients exhibit seizures, stroke, flaccid paraparesis, corticospinal weakness, and even coma. Moreover, the neurological manifestations can occur independently of the respiratory system. In conclusion, SARS-CoV-2 infection can cause multiple neurological syndromes in a more complex presentation. Therefore, this review elucidated the involvement of the nervous system in SARS-CoV-2 infection and will hopefully help improve the management of COVID-19.

## INTRODUCTION

The severe acute respiratory syndrome coronavirus 2 (SARS-CoV-2) is the name that was attributed to the virus formerly known as the novel coronavirus, which is a newly emerged zoonotic virus that causes the coronavirus disease 2019 (COVID-19) [1]. SARS-CoV-2 infection was first reported in Wuhan, Hubei Province, China, on December 29, 2019, where four cases of an acute respiratory distress syndrome of unknown etiology were linked to a local Huanan South China Seafood Market; since then, this virus has caused a global pandemic [2]. In general, coronaviruses are common in animals, with some, namely, HCoV-229E, HCoV-OC43, HCoVNL63, and HCoV-HKU1, affecting humans and generally causing a mild respiratory illness [3,4]. However, several coronaviruses have caused outbreaks in the past two decades, including the severe acute respiratory syndrome coronavirus (SARS-CoV) outbreak of 2002/2003, which affected 8422 people across 26 countries and caused 916 deaths (i.e., a mortality rate of 11%) [5,6], as well as the Middle-East respiratory syndrome coronavirus (MERS-CoV) outbreak of 2012/2013, which affected 1386 people and caused 587 deaths [7]. Similar to that observed for SARS-CoV, patients infected by MERS-CoV suffered from pneumonia followed by severe acute respiratory distress syndrome and multiple organ failure.

SARS-CoV-2 infection results in a syndrome of various systemic and respiratory symptoms such as dry cough, breathing difficulty, fever, and fatigue, which sometimes can be critical by causing severe pneumonia and cardiorespiratory failure and requiring specialized management in intensive care units [8,9]. Recently, it has been documented that, in addition to systemic and respiratory symptoms, some patients with COVID-19 develop neurological symptoms. These symptoms include headache, altered consciousness, anosmia, and paresthesia, among many others [10]. In addition, an increasing number of cases of patients with COVID-19 that develop encephalopathy [11] and Guillain–Barré syndrome (GBS)-like manifestations is being reported [12,13]. Considering the ongoing global pandemic of COVID-19 and the descriptions of neurological manifestations in SARS-CoV-2 infection, it is necessary to alert clinicians regarding the high likelihood of nervous system involvement in this disease.

## MATERIALS AND METHODS

**FIGURE 1:**
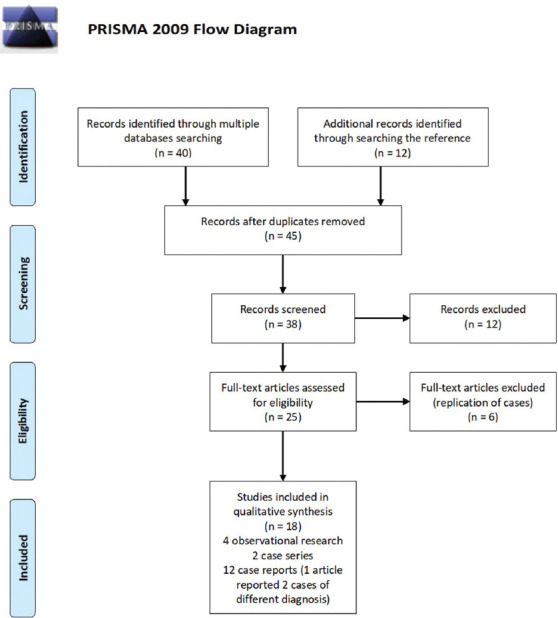
Flow chart of the literature search process according to the preferred reporting items for systematic reviews and meta-analyses (PRISMA) guidelines.

An exhaustive search of scientific publications (original articles on relevant experimental and observational studies, case series, and reports) was conducted using the following online databases/online search engines: Google Scholar, Web of Science, Scopus, Medline/PubMed, bioRxiv, medRxiv, and ChemRxiv, as well as CNKI and WanFang Data (which are the two primary databases for biomedical research in mainland China). The search terms used were: “Neurological manifestations of COVID-19”, “Neurological manifestations of novel coronavirus 2019”, “Neurological manifestations of SARS-CoV-2” “Neurological complications of COVID-19”, “Neurological complications of coronavirus 2019”, and “Neurological complications of SARS-CoV-2.” All relevant articles were analyzed for a possible neurological syndrome related to COVID-19. These articles had either been published or were in preprint from January 1, 2020, to April 25, 2020. Fifty articles were organized according to the search words. The reference lists of these articles were also searched and analyzed for additional findings or reports related to nervous system involvement in COVID-19. A flow chart of the search process is provided in Figure 1. These articles were then carefully filtered for their relevance based on the following selection criteria: Diagnosed cases of COVID-19, cases with the neurological manifestation of neurological syndrome, a clear description of the clinical cases, and studies carried out in animal models or diagnosed human patients with SARS-CoV-2 infection involving the central nervous system. The relationship between COVID-19 and the nervous system was explored and a brief review was then performed.

## RESULTS

We reviewed more than 50 scientific articles on SARS-CoV-2 infection with relation to the nervous system that were either published or pre-published (accepted for publication and in preprint) in the past 4 months, including case reports, case series, experimental studies, observational studies, review articles, and letters to the editor. We look into the details of the neurological manifestations or complications of COVID-19 disease and reviewed the possible mechanism of viral transmission to the nervous system. After considering the selection criteria, 13 case reports, two case series, and four observational studies were included in the study.

### Neurological manifestation of SARS-CoV-2 infection

Anosmia and ageusia are among the neurological symptoms most commonly reported by patients with COVID-19. Eliezer et al. [14] reported a case of a young female who lost her sense of smell without any nasal obstruction. A computed tomography (CT) scan and magnetic resonance imaging (MRI) of the nasal cavity showed only bilateral inflamed olfactory clefts without any anomalies in the olfactory bulb and tract. Spinato et al. [15] conducted a study in Italy, in which they called 202 patients with COVID-19 through the phone to determine if they had any neurological symptoms. One hundred and three of these patients (64.4%) complained of anosmia and ageusia, among whom 24, 46, and 54 individuals developed thee symptoms prior, concomitantly, and after fever and cough, respectively, and six patients only had anosmia and ageusia without any fever. Anosmia and ageusia were also reported by Mao et al. [10], who performed an observational study of 214 patients with COVID-19. In that study, 78 (36.4%) patients developed neurological manifestations, which included CNS symptoms (such as dizziness, headache, and loss of consciousness) and peripheral nervous system (PNS) symptoms (such as anosmia and ageusia). In addition, 23 patients developed rhabdomyolysis of varying severity. Suwanwongse et al. [14] later reported an interesting case of rhabdomyolysis in an elderly patient with COVID-19 whose presentation was only bilateral lower-limb weakness without any fever or respiratory symptoms. Mao et al. [10] also reported six patients who developed ischemic stroke. This finding raises a concern regarding the inadequacy of the information available on cerebrovascular involvement in COVID-19 [16]. Therefore, Li et al. [17] carried out an observational study of 221 COVID-19 patients aimed at identifying the presence of cerebrovascular disease. The authors found that 11 (5%) of these patients developed acute ischemic stroke, one (0.5%) patient developed cerebral venous sinus thrombosis, whereas one other 1 (0.5%) patient developed cerebral hemorrhage. The majority of the patients with the cerebrovascular disease were elderly individuals, and 84.6% of them presented with severe SARS-CoV-2 infection. Furthermore, five of the 13 patients with the cerebrovascular disease died. Subsequently, Oxley et al. [18] published a case series of five young patients with COVID-19 who developed large vessel ischemic stroke, whereas intracranial bleeding was observed by Sharifi-Razavi et al. in one patient with this disease (2020) [19].

An observational study of 58 patients with COVID-19 who were admitted to a hospital in France for severe acute respiratory distress syndrome reported by Helms et al. [20] found that 39 (67%) of these patients developed diffuse corticospinal tract signs with enhanced tendon reflexes, ankle clonus, and bilateral extensor plantar reflexes. Three patients had a subclinical ischemic stroke, which was noted on MRI of the brain. That study also found that 15 (33%) of the 45 patients who were discharged had a dysexecutive syndrome such as inattention, disorientation, or poorly organized movements in response to commands. Another neurological syndrome that was frequently reported in patients with SARS-CoV-2 infection was GBS. Zhao et al. [12] reported the case of an elderly female in China who presented with sudden bilateral lower-limb weakness before developing low-grade fever and dry cough. She was treated successfully with an immunoglobulin infusion (IVIg) and lopinavir/ritonavir and was discharged well. A case series of five patients with COVID-19 and GBS from Italy was reported by Toscano et al. [13]. In that report, all five patients had respiratory symptoms before the onset of weakness, and only three of them had a fever. All of these patients were treated with IVIg. Two of the patients remained in the intensive care unit and required mechanical ventilation, two patients were discharge for physiotherapy with some recovery, and one patient was able to walk independently at discharge. There was also a case of Miller–Fischer syndrome that occurred in COVID-19 patients reported in the literature. Gutiérrez-Ortiz et al. [21] described a male patient who had a fever, anosmia, and ageusia for a few days before developing ataxia, vertical diplopia, and generalized areflexia. His cerebrospinal fluid (CSF) exhibited cytoalbuminological dissociation, and he was positive for the GD1b-IgG antibody. He was treated with IVIg and discharged well. In the same article, the authors also described another interesting case of COVID-19, who developed isolated multiple cranial neuropathies, which can be a mild spectrum of Miller–Fischer syndrome. The patient had a low-grade fever for 3 days before he developed diplopia. His CSF analysis was normal. His anti-ganglioside antibody level was not assessed, and he was not admitted because of logistics issues associated with an overcrowded hospital. He was noted to have recovered spontaneously during a telemedicine follow-up.

Three case reports described the development of acute encephalitis in patients with COVID-19 from China [22], Iran [23], and Japan [24], respectively. Two of the cases were young patients aged 24 and 30 years, while the other patient was 56 years old. All three patients presented with fever, cough, and impaired consciousness, while two of them developed generalized-onset tonic–clonic seizures. The outcome of two of these patients was good, while that of the third-one was not mentioned. In a more extreme scenario, cases of acute necrotizing encephalopathy [25] and acute disseminated encephalomyelitis [26] were reported. Both of these patients presented with altered mental status and were treated with IVIg. Zhao et al. [27] reported a case of acute transverse myelitis in an elderly patient with COVID-19. He was treated with a 7-day course of IVIg and some recovery of his muscle strength was recorded. A brief summary of all the articles selected for this review is provided in Table 1.

**TABLE 1:**
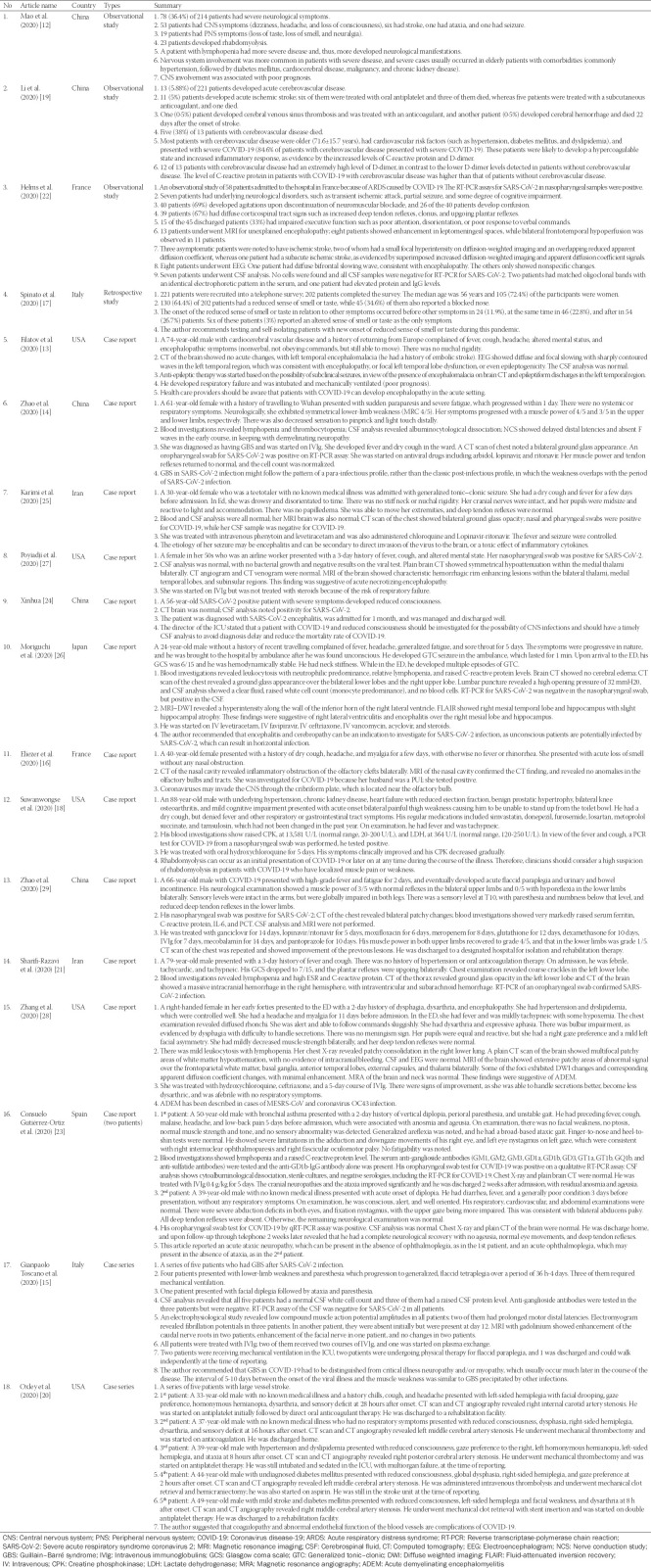
Brief clinical summaries of the selected articles, which included 13 case reports, two case series, and four observational studies

### Possible mechanism of SARS-CoV-2 invasion into the nervous system

SARS-CoV-2 is believed to invade the CNS from the peripheral nerve terminals through neural pathways, such as the olfactory nerves. The olfactory nerves have a unique anatomical organization that provides a channel between the olfactory bulb, located near the nasal cavity, and the brain [28]. Furthermore, there is evidence of the presence of SARS-CoV-2 genetic material and proteins in samples from nervous system tissues [29]. This suggests that the virus can directly invade and damage the nervous system. The virus also triggers an exaggerated immune response that attacks the nervous system, as observed in multiple cases of patients with severe COVID-19 who developed a cytokine storm syndrome, which is a hyperinflammatory state characterized by a fulminant hypercytokinemia leading to life-threatening multiorgan failure [30]. Among these cytokines, interleukin-6 (IL-6) is elevated in patients with COVID-19. The level of IL-6 is positively correlated with the severity of COVID-19 symptoms [31]. Moreover, IL-6 is an important pro-inflammatory mediator that is potentially responsible for the activation of immune cells in the brain and injury of the brain tissue [32]. Another plausible hypothesis is that the ectodomain of the spike protein of SARS-CoV-2 has a high binding affinity to the angiotensin-converting enzyme 2 (ACE2) receptor [33]. The ACE2 receptors are expressed abundantly in the capillary endothelium of multiple parts of the human body, including the brain, to which the virus may gain access through the blood-brain barrier and damage the nervous system [34]. It has been reported that glial cells and neurons in the brain also express ACE2 receptors [35], which renders them a potential target of SARS-CoV-2. In addition, as ACE2 is a vasoconstrictor and has a pro-inflammatory effect [36], it is also possible that ACE2 involvement in the brain during COVID-19 infection leads to autoregulatory disruption and blood pressure spikes, resulting in arterial wall rupture, which may be the pathophysiology underlying the intracranial bleeding observed in SARS-CoV-2 infection [19]. Conversely, ischemic stroke is not uncommon in patients infected with SARS-CoV-2. This may be explained by the fact that SARS-CoV-2 causes endothelial dysfunction [18] and increases coagulability, as evidenced by the increased levels of C-reactive protein and D-dimer observed in these patients [17,18,37]. Another hypothesis proposes that SARS-CoV-2 also affects the nervous system in a simpler way, that is, the severe hypoxia resulting from pneumonia and acute respiratory distress syndrome causes cerebral edema and ischemic stroke [38].

## DISCUSSION

SARS-CoV-2 belongs to the genus of beta-coronaviruses, which are zoonotic viruses that can infect both animals and humans. It is the seventh type of coronavirus to affect humans and the third-one to cause a global pandemic. Infection with SARS-CoV-2 can cause a typical systemic and respiratory clinical syndrome that includes symptoms such as fever, cough, shortness of breath, myalgia, and fatigue. Other clinical presentations that are consider non-typical of this virus are diarrhea, anorexia, conjunctival congestion, nausea, and vomiting, among many others [39]. About 20-30% of patients may exhibit progression to more critical conditions, such as acute respiratory distress syndrome, septic shock, disseminated intravascular coagulation, acute heart failure, and acute kidney injury [40]. The incubation period for SARS-CoV-2 infection is about 2-14 days [41]. The transmission has been confirmed to occur rapidly from human to human, and it is thought to occur through direct contact with respiratory droplets from an infected individual [42]. As a result of a lack of awareness regarding infection control, especially in hospitals and international airports, the infection with this virus has spread rapidly across borders and has caused a massive global pandemic [43]. To date, there are more than 6 million confirmed cases of COVID-19, and more than 350,000 deaths have occurred in more than 150 countries around the world.

As shown in previous studies of SARS-CoV and MERS-CoV, beta-coronaviruses are neurotropic and exhibit neurovirulent properties [44]. Since SARS-CoV-2 has 79% and 52% genetic similarity with SARS-CoV and MERS-CoV, respectively [45], it is possible that it possesses similar properties and is capable of infecting the nervous system. Multiple hypotheses have been put forward regarding the possible mechanisms through which SARS-CoV-2 affects the nervous system. These include direct invasion of SARS-CoV-2 into the nervous system, as evidence by the discovery of the viral protein in the CSF of a patient with COVID-19 who was suspected of developing encephalitis [24,30]. The route of invasion can be either through retrograde movement through the olfactory nerve [29] or the hematogenous route, as the presence of ACE2 receptors in the brain may facilitate the movement of the virus through the brain’s circulation [35,36]. Another possible route of invasion is through the hyperactivation of the host immune response and to the triggering of a hyperinflammatory state with multiorgan failure, in a condition called cytokine storm syndrome [31]. This is only to name a few of the many suggested possible mechanisms through which SARS-CoV-2 can affect the nervous system. However, the exact mechanism is still not fully understood, and further studies are needed to explore this subject.

There is evidence showing the association between SARS-CoV-2 infection and nervous system involvement. This association can occur regardless of the involvement of the respiratory system. Moreover, it is observed more commonly among patients who are admitted to the hospital with severe illness and in elderly patients with multiple comorbidities [10]. The neurological manifestations of COVID-19 disease can be broadly divided into CNS and PNS symptoms. The most commonly reported CNS presentations to include impaired consciousness, headache, dizziness, confusion, and agitation 10,20]. Regarding PNS involvement, loss of taste, loss of smell, and neuralgia are the commonly reported symptoms [14-16]. Furthermore, some patients develop more sinister neurological syndromes such as GBS [12,13], acute ischemic stroke [10,17,18], intracranial haemorrhage [19], acute myelitis [27], acute encephalitis [22-24], acute necrotizing encephalopathy [25], and acute disseminated encephalomyelitis [26].

Another major concern regarding the involvement of the nervous system in SARS-CoV-2 infection is the possibility of long-term or permanent neurological disabilities. This is because the neurological syndrome can last longer than the lung infection itself [46]. Moreover, other types of coronaviruses are linked to the development of CNS dysfunction, such as multiple sclerosis [47]. Therefore, further studies are deemed necessary to elucidate the prognosis and potential reversibility of the neurological syndromes of COVID-19, as well as the impact of SARS-CoV-2 infection in promoting other neurological diseases, such as multiple sclerosis.

This study had limitations, as most of the articles analyzed here were case reports, and only a few research articles pertained to observational studies from a single center with a very limited number of cases. Finally, this article used mainly descriptive analyses to review and summarize the clinical cases of COVID-19 with nervous system involvement.

## CONCLUSION

Based on the evidence gathered from the scientific literature, this review raises the possibility of nervous system involvement in COVID-19. Therefore, it would be prudent to evaluate all patients with COVID-19 for neurological symptoms, and to rule out SARS-COV-2 infection in any patient presenting with unusual neurological symptoms, to improve the prognosis of COVID-19 by delivering appropriate management in a timely fashion.
